# Challenges in the provision of kidney care at the largest public nephrology center in Guatemala: a qualitative study with health professionals

**DOI:** 10.1186/s12882-020-01732-w

**Published:** 2020-02-28

**Authors:** David Flood, Katharine Wilcox, Andrea Aguilar Ferro, Carlos Mendoza Montano, Joaquin Barnoya, Pablo Garcia, Randall Lou-Meda, Peter Rohloff, Anita Chary

**Affiliations:** 1 National Clinicians Scholars Program, Division of Hospital Medicine, University of Michigan, Ann Arbor, MI USA; 2 Wuqu’ Kawoq | Maya Health Alliance, Tecpán, Guatemala; 3 Weill Cornell School of Medicine, New York, USA; 4 Institute of Nutrition of Central America and Panama (Instituto de Nutrición de Centroamérica y Panamá, INCAP), Guatemala City, Guatemala; 5 Unit for Cardiovascular Surgery (Unidad de Cirugía Cardiovascular de Guatemala, UNICAR), Guatemala City, Guatemala; 6 Institute of Research and Higher Studies in Health Sciences (El Instituto de Investigación y Estudios Superiores en Ciencias de la Salud, IECIS), Rafael Landívar University, Guatemala City, Guatemala; 7 Division of Nephrology, Stanford University, Palo Alto, USA; 8 Foundation for Children with Renal Disease (Fundación para el Niño Enfermo Renal, FUNDANIER), Guatemala City, Guatemala; 9 Department of Medicine, Department of Global Health Equity, Brigham and Women’s Hospital, Boston, USA; 10 Department of Emergency Medicine, Massachusetts General Hospital, Boston, USA

**Keywords:** Qualitative research, Global kidney care, Global health, End-stage kidney disease, Burnout, Guatemala

## Abstract

**Background:**

Chronic kidney disease (CKD) is increasing worldwide, and the majority of the CKD burden is in low- and middle-income countries (LMICs). However, there is wide variability in global access to kidney care therapies such as dialysis and kidney transplantation. The challenges health professionals experience while providing kidney care in LMICs have not been well described. The goal of this study is to elicit health professionals’ perceptions of providing kidney care in a resource-constrained environment, strategies for dealing with resource limitations, and suggestions for improving kidney care in Guatemala.

**Methods:**

Semi-structured interviews were performed with 21 health professionals recruited through convenience sampling at the largest public nephrology center in Guatemala. Health professionals included administrators, physicians, nurses, technicians, nutritionists, psychologists, laboratory personnel, and social workers. Interviews were recorded and transcribed in Spanish. Qualitative data from interviews were analyzed in NVivo using an inductive approach, allowing dominant themes to emerge from interview transcriptions.

**Results:**

Health professionals most frequently described challenges in providing high-quality care due to resource limitations. Reducing the frequency of hemodialysis, encouraging patients to opt for peritoneal dialysis rather than hemodialysis, and allocating resources based on clinical acuity were common strategies for reconciling high demand and limited resources. Providers experienced significant emotional challenges related to high patient volume and difficult decisions on resource allocation, leading to burnout and moral distress. To improve care, respondents suggested increased budgets for equipment and personnel, investments in preventative services, and decentralization of services.

**Conclusions:**

Health professionals at the largest public nephrology center in Guatemala described multiple strategies to meet the rising demand for renal replacement therapy. Due to systems-level limitations, health professionals faced difficult choices on the stewardship of resources that are linked to sentiments of burnout and moral distress. This study offers important lessons in Guatemala and other countries seeking to build capacity to scale-up kidney care.

## Introduction

Chronic kidney disease (CKD) incidence and prevalence are rising worldwide [1]. The majority of the global CKD burden is in low- and middle-income countries (LMICs) [2]. However, there is wide variability in access to kidney care treatments such as renal replacement therapy (RRT) for end-stage kidney disease (ESKD) in LMICs [3, 4]. These factors have led kidney disease to be described as a neglected chronic disease [5].

Guatemala is an upper-middle income country in Central America with a population of 16.3 million people [6]. The disease epidemiology in Guatemala includes a rising prevalence of adult non-communicable diseases alongside a persistent burden of maternal and child disorders, especially child stunting. The Guatemalan constitution guarantees universal health coverage, without discrimination, to all citizens [7]. This guarantee has not been realized in practice [8]. Given the country’s very low per-capita public health spending relative to peers [9], the health system has struggled to expand access to both comprehensive primary care and specialty care technologies such as cancer treatment and dialysis [10]. The government health system is also marked by disparities in health care access and allegations of abuse within the country’s large indigenous Maya population [11].

In Guatemala and throughout the Latin American region, traditional risk factors such as diabetes and hypertension are the primary drivers of progression to ESKD and renal disease-related mortality [12–14]. Additionally, an emerging clinical entity of chronic kidney disease of unknown etiology (CKDu, also described as “Mesoamerican nephropathy”) in agricultural workers without traditional risk factors may contribute to the rising CKD burden in Central America [15, 16]. There is wide variability in access to kidney care in Latin America due to multiple factors including health system fragmentation and underfunding [17]. Prevalence rates of dialysis and kidney transplantation are very heterogeneous in the Latin American region, suggesting that access is a critical issue [14].

In Guatemala, nephrology care for people with CKD, including those with ESKD requiring RRT, is primarily delivered by two institutions: the Social Security Health System (IGSS) and the Ministry of Health, which operates both the Foundation for Children with Renal Disease (FUNDANIER) and the National Center for Chronic Renal Disease (UNAERC). Approximately 8% of the Guatemalan population has access to private insurance [18], so CKD services are less commonly delivered in the private sector. IGSS is an employer-based health insurance system available to 18% of the overall population [18] that provides RRT to approximately 3700 individuals [19]. FUNDANIER is a pediatric nephrology center that serves approximately 500 children with RRT including renal transplantation [20].

The present study was carried out with health professionals at UNAERC, the public-sector institution responsible for providing dialysis to uninsured Guatemalan adults. UNAERC has experienced rising patient volume in recent years. The institution’s dialysis census increased from fewer than 2000 patients in 2008 [21] to over 5000 in 2019 [22]. Guatemala possesses among the world’s highest proportion of people with ESKD using peritoneal dialysis [23], and 60% of UNAERC dialysis patients use continuous ambulatory peritoneal dialysis (CAPD). Automated peritoneal dialysis is rarely used in Guatemala. UNAERC is headquartered in Guatemala City with a satellite dialysis unit in Escuintla, a south-Pacific department with a high CKDu prevalence [16].

As an institution, UNAERC faces systemic challenges. First, the institution’s budget outlays have not risen in proportion to meet the population-level increase in demand for RRT [24]. Second, UNAERC serves a diverse and impoverished population with socioeconomic, geographic, and linguistic barriers to care [25, 26]. Finally, the institution resides in a complex, high-pressure political environment [27, 28].

This qualitative study explores the work of health professionals at UNAERC. The goal of the study is to elicit health professionals’ perceptions of providing kidney care in a resource-constrained environment, strategies for dealing with resource limitations, and suggestions for improving kidney care in Guatemala.

## Methods

### Study design

We conducted qualitative semi-structured interviews from November to December 2018 with a purposeful sample of 21 health professionals from UNAERC in Guatemala City (Table 1). The research team identified and contacted participants through referrals from administrative assistants at UNAERC. As detailed below, a codebook of themes was generated as interviews were completed such that recruitment continued until data saturation was achieved.

**Table 1:** Participants’ Roles at UNAERC

Provider Type	No. (%)
Nurses	4 (19%)
Physicians	7 (33%)
Administrators	2 (10%)
Allied health professionals (technician, psychologist, nutritionist, social worker)	8 (38%)
**Total**	**21**

### Data collection

Semi-structured interviews lasting 20–40 min were performed in private offices of UNAERC. All interviews were conducted in Spanish by a team of trained interviewers [AAF, ED, KW] and supervised by a senior author with expertise in qualitative methods [AC]. Interviews focused on challenges faced by providers, strategies for reconciling high demand for services with limited resources, and suggestions for improvement. (See Additional file 1 for interview guide.) Interviews were recorded with consent from each participant and transcribed verbatim by a native Spanish-speaker [AAF]. Transcripts served as the basis for data analysis.

### Data analysis

Qualitative data from interviews were analyzed using an inductive approach, allowing dominant themes to emerge from interview notes. We developed and refined a codebook in successive rounds of review of transcripts. Two researchers independently coded data into thematic categories using the qualitative data analysis program NVivo [KW, DF]. The senior author [AC] reviewed all coded data and guided team members in collective review to reach consensus about the application of codes.

### Institutional context and ethics

This research was performed through a collaborative capacity-building project for CKD research in Guatemala among the following institutions: the Institute for Nutrition of Central America and Panama (INCAP), the Unit for Cardiovascular Surgery (UNICAR), and Wuqu’ Kawoq | Maya Health. INCAP and UNICAR are leading research and cardiovascular institutes in Central America, respectively, and both are based in Guatemala City. Wuqu’ Kawoq | Maya Health is a non-governmental organization based in Tecpán, Guatemala that provides health services, including services for non-communicable diseases such as CKD, in rural indigenous Maya communities in Guatemala. This study was reviewed and approved by the Institutional Review Boards of INCAP, Wuqu’ Kawoq | Maya Health, Partners Healthcare (Boston), and UNAERC. All participants were above the age of 18 and provided verbal informed consent. Participants were not asked to provide a signed consent form in order to avoid creating a record linking their identities to study participation. The verbal consent process was approved by the three Institutional Review Boards above. We followed the Standards for Reporting Qualitative Research (SRQR) guidelines in reporting this research [29].

### Reflexivity in the research process

Within qualitative research, reflexivity is the process by which researchers consider how their social positions as well as personal values and interpretations may influence research [30]. Study team members hale from both Guatemala [AAF, JB, ED, PG, RL, CMM] and the United States [AC, DF, PR, KW]. All team members had prior personal and professional experience studying health disparities or providing health care in Guatemala. Interviewers in this study were health professionals [AC, ED, KW] or social scientists [AAF, AC] without affiliations to UNAERC. KW, DF, and AC, all US-trained physicians with clinical experience in rural Guatemala, were primarily responsible for data analysis. Given their experiences of health resource scarcity were shaped by multiple contexts, they solicited feedback on data analysis from UNAERC health professionals through a written report and an oral presentation. UNAERC health professionals agreed with the major themes and interpretation of data presented herein.

## Results

Themes emerged in three primary areas: (1) challenges related to resource limitations and allocation, (2) emotional challenges, and (3) suggestions for improvement (Table 2).

**Table 2:** Representative quotes

Theme	Representative quote
Resource limitations and allocation	“You have 200 patients, and you have 75 machines to use, so how do you choose? So the only thing that we can do, because we cannot evaluate all the patients at the same time, the only thing we can do is look at objective data, such as times of the last hemodialysis, see where the patient comes from, the age of the patient, if he is elderly or is a kid, and if he already had peritoneal dialysis [i.e., a patient previously on peritoneal dialysis who is no longer able to continue with the modality], because if he already had peritoneal dialysis, he has no other option.” -Physician “Our patients are standing all the time waiting for their consultations, and I cannot even say, ‘Sit here, because I have a free chair,’ because I only have two chairs.” -Allied health professional “The limits on time and space are so frustrating, and mainly the resource limitations. Because we would like to give them all their medicines, but we truly cannot. We only give you what is truly within our reach. And there are many people who do not get any medicine because they do not have the resources to obtain them.” -Allied health professional
Emotional challenges	“The worst challenge, from my point of view, is the fact that the patient is not receiving their three treatments a week. Why? I am nothing more than a little soldier in the ranks of combat. I know that the patient will come to me intoxicated, uremic, with cardiac deficiencies—grandparents of 70 years that I know I am going to connect to the machine, and I know that at that moment they are going to have a complication.” -Allied health professional “For me it is a challenge every day to keep the patient alive, because the patient enters breathing, but [if something] goes wrong … The fact is that the machine is not magic. The machine is not going to change your life overnight.” -Allied health professional “Many times we feel disappointed as providers. We experience what is called ‘burnout’—and we are already burned out—but then, we move on.” -Physician “[I see it] as a challenge … to meet the needs of our patients—the needs of our patients from the medical point of view. I always make a half-joke with the patient who arrives, ‘What are your problems? Please tell me they are not economic, anything but economic, because that I will not be able to solve for you.’” -Physician
Suggestions for improvement	“You see how crowded we are, right? It would make me happy to have a larger place where we could better serve patients. First physical space, is what we need more of.” -Administrator “Sure, we have to expand services, we have to decentralize, but...what we have to do is not only open dialysis centers and that’s it. In other words, to do that is not to do nothing—it would only be for those who are sick, but we will not help the ones who will come later. So that’s what should be done: prevent, treat the sick, and transplant.” -Physician “Have more people trained in kidney nutrition, and psychologists, because at UNAERC there is a single psychologist to see the large number of patients, and the truth is that that is not enough. She was being asked to treat patients and that is impossible, because with each patient it would take at least an hour, what, at least half an hour, and the truth is that with the large list of people, that is impossible...Also the issue of social workers, because there are two, but that is not enough for all the problems of the patients.” -Physician

### Resource limitations and allocation

#### Hemodialysis supply and demand

All participants described that the demand for hemodialysis greatly exceeds UNAERC’s capacity. The institution has 60 HD machines in Guatemala City and provides 240 HD sessions daily, divided in four shifts. UNAERC would have to provide 630 HD sessions per day to give thrice weekly sessions to the current census of patients. Interviewees commonly described limitations in quantity of HD machines, physical space, and personnel that precluded them from accommodating the volume of patients in need. Indeed, interviewees reported that institutional capacities are already stretched thin with the current daily patient census. Staff regularly turn away patients who are scheduled for HD due to resource shortages and the need to accommodate clinical emergencies.

Interviewees described several strategies for reconciling high demand for HD with limited institutional resources. First, eight subjects stated the institution’s first response is to decrease the frequency and duration of HD treatments. Most UNAERC patients receive HD every 7–10 days rather than three times a week. Each HD session runs for a set three hours rather than for a duration of time adjusted based on dialysis adequacy for each individual, allowing a fourth shift to be added to the day. Recognizing that many patients travel from rural areas, providers assign these patients to the morning or earlier shifts to facilitate return travel and those who live in or close to the capital to afternoon or later ones.

Second, eight providers described evaluating patients’ clinical presentation to prioritize those with the most urgent need for HD on a daily basis. Patients are expected to arrive with completed labwork from an outside facility, and those who do not arrive with this documentation may be allowed entry to obtain labwork at UNAERC or turned away, depending on how busy the institution is each day. Those with uncontrolled hypertension, electrolyte abnormalities, and uremia are prioritized for dialysis. As one physician explained:

In the first place, hospitalized patients have priority. Every day, patients who are sent here from other hospitals have priority and we leave spaces open for them. Priority number two are the emergent patients, those who come in bad shape, they feel horrible, they are really in poor condition and they need urgent dialysis. And then we also give priority to those patients who were already [i.e., previously initiated then unable to continue] on peritoneal dialysis, because they tried the alternative and for one reason or another they could not continue.

Providers recognized that some patients, particularly those with limited literacy, have difficulty understanding the paperwork requirements—identification, referral letter, and labwork—that they must present for clinical evaluation before each dialysis session. For example, one technician stated that “normally they only come with [one] sheet of paper”—sometimes, identification or a referral letter only—but can only be called for HD “when they gather the rest.”

Fifteen providers described a third institutional strategy for reconciling demand for HD with resource limitations: encouraging new patients to enroll in CAPD, rather than HD. Providers presented CAPD as ideal for Guatemala’s large rural population, for whom frequent travel to Guatemala City presents both direct and indirect economic costs, such as transportation costs and days’ wages lost in travel, respectively. Nurses involved in new patient enrollment reported that in addition to educating patients about the differences between HD and CAPD, they inform patients about the realities of overcrowding and wait times for HD to help motivate them to consider CAPD. As one technician related:

When they enroll in the institution, patients are given the option of peritoneal or hemodialysis, but in fact they are asked to opt for peritoneal due to the number of patients we have to manage in hemodialysis…At first consultation, they are given a talk about what the disease is, and they are told the benefits of peritoneal and hemodialysis, but they are also told that in hemodialysis there is no space.

A team assesses patients’ eligibility for CAPD, based on clinical data and a their ability to dedicate a clean room in their house to CAPD. Those who enroll in CAPD have a peritoneal catheter placed at UNAERC, receive free CAPD supplies delivered to their homes, and attend regular outpatient consultations at UNAERC. Approximately 3000 patients are currently enrolled in CAPD. Overall, staff expressed that resource limitations are not as much of a problem with CAPD as with HD, in part due to subsidized materials from the Ministry of Health.

#### Patient volume and staffing

Most interviewees (*n* = 14, 67%) described the challenges of providing outpatient clinical, nutrition, psychology, and social work care to a large volume of patients on a daily basis. Providers reported seeing between 30 and 50 patients in 8 h, which they described as “tiring,” “impossible,” and “too great a burden.” Six providers cited a need to decrease the amount of time spent with and the quality of care provided to each patient. As a psychologist stated:

My job is to talk to people, and there are many patients who have very big problems. And I have to say, “Sorry, but we cannot [go on talking], because I have a line of people waiting for me.” So I try to optimize the time or the quality of time that I can offer to each patient… Many people ask me, “Why do not you give 30 minutes to each patient?” It's impossible, I only have 8 hours to be here.

Similarly, a physician stated:

I cannot tell a patient who is hypertensive, or who comes with volume overload, that we will give him the consultation for tomorrow, no. You definitely have to take care of it. What happens is that you cannot provide care to the quality that one should give…. For example, if someone comes with peritonitis, and one cannot even give him ten minutes—and the patient wants to talk, “Look doctor, I have this doubt,” and I have no time.

#### Medications

Twelve providers described the lack of essential dialysis-related medications as a serious institutional challenge. UNAERC does not have the budget to provide patients erythropoietin, iron, phosphate binders, and anti-hypertensive agents free of charge. Providers expressed awareness that many patients cannot afford these medications. The institution sometimes receives donations, which clinicians distribute based on severity of clinical need. However, as one physician explained, when overwhelmed with great need from all patients, providers do not always adhere to clinical protocols for resource allocation:

We got a donation of 24,000 ampules of erythropoietin 4000 IU, which helped us for a while. What we did was we gave it to those on CAPD who needed it the most. We gave it to patients with hemoglobin under 8, and the others had to buy their erythropoietin. Sometimes I would skip that rule and give it to everyone I could, because the truth is that the need is too great.

### Emotional challenges

One of the most common themes that emerged from interviews was the scope of emotional challenges faced by providers in treating patients at UNAERC. Nineteen of the 21 interviewees described feelings of guilt, disempowerment, and sadness related to turning patients away and not being able to provide optimal treatments. For example, one nurse described:

In my day to day work, the worst is to tell the patient, “there is no machine.” The worst is to tell the patient, “today I cannot treat you.” … It is the worst feeling to not able to meet the patient's needs. Or to listen to a mother who is crying because she needs treatment for her son and cannot get it. Or listening to a patient who comes from far away, “Please help me, please dialyze me today because I cannot afford to come back another day.” And I cannot help him.

One physician, who sees approximately 50 patients daily, reflected with guilt and disappointment upon poor patient-provider relationships that result from having insufficient time with each patient:

The patient says “they did not take care of me,” and he’s right. We mistreated him. But it is not an abuse that we do because we want to do it. I mean, I’m not mistreating you because I’m screaming at you, I’m saying foul words, or I’m being pejorative with you. Rather, I’m mistreating you because you demand a better quality consultation. Maybe you want to talk about your medical or psychological problems, or social problems that you want to resolve and I do not have time to listen to you…Then it becomes an indirect abuse, but it is still abuse.

Interviewees across health professions described feelings of exhaustion and devaluation, with four physicians specifically using the English term “burnout.” Providers reported physical depletion associated with relentless patient volume and not having time to eat or use the bathroom while at work. Interviewees described a high emotional toll of dealing with day-to-day patient suffering. As one nurse stated:

The emotional charge we handle—of caring for the patient and seeing that he is in bad shape, he is depressed—it really gets to us.

Interviewees noted that modest salaries, delayed wage payments, and inability to take time off for vacation contributed to feeling “resigned” and “desperate” about work. One senior physician recognized the importance of being able to take breaks both at and from work to avoid exhaustion, but noted that institutional conditions did not regularly allow for this. Three interviewees mentioned that four physicians had recently left the institution, which staff partly attributed to demanding working conditions.

Notably, despite emotional burdens, ten interview subjects expressed feelings of vocation and job satisfaction. For example, one technician reported achieving a sense of importance in her daily work:

I think that UNAERC is a place where God works miracles. Every day we do miracles, and maybe we do not notice them but really seeing a person who comes back to life again is wonderful … that's something that completely fills you up.

Other health professionals similarly reported resilience stemming from witnessing the beneficial effects of treatment on individual patients or appreciating one’s own role as a clinician with a unique skillset to work with the underserved.

### Suggestions for improvement

Table 3 details common themes that emerged as suggestions for improvement of services provided by UNAERC. All 21 interviewees discussed a need for an increased institutional budget. Administrators specifically noted a need for public health and policy leaders to prioritize CKD. The majority of providers (*n* = 14, 67%) cited a need for physical expansion of UNAERC’s facility in Guatemala City, both to accommodate larger consult and waiting areas as well as an increased number of dialysis machines. About half of providers (*n* = 12, 57%) advocated for decentralizing UNAERC by opening satellite facilities in other areas of the country. Interviewees pointed to UNAERC’s single satellite facility on the south-Pacific coast, which helps reduce rural patients’ travel burden and reduces patient volume in Guatemala City.

**Table 3:** Suggestions for improvement

Theme	No. (%)
Increase budget	21 (100%)
Physical expansion	14 (67%)
Decentralization with satellite sites	12 (57%)
Hire more personnel	9 (43%)
Invest in pre-dialysis and preventive health services	8 (38%)
Provide essential dialysis-medications at no cost	7 (33%)
Develop transplant services	5 (24%)

Less frequently, providers mentioned hiring more personnel (*n* = 9, 43%), investing in pre-dialysis and preventive services (*n* = 8, 38%), improving UNAERC’s ability to provide patients essential dialysis-related medications at no cost (*n* = 7, 33%), and developing increased access to services (*n* = 5, 24%).

## Discussion

This qualitative study describes the experiences of health professionals who provide kidney care to a large, underserved population at a public-sector nephrology center in Guatemala. Key themes were the strategies health professionals employed to reconcile high demand with limited resources, emotional challenges arising from caring for a high clinical volume, and potential suggestions for improvement. Our findings have implications for the delivery of kidney care in this context and similar settings globally [3, 17].

First, this study outlines how systems-level constraints at a nephrology center become actualized at the level of the clinician-patient encounter. For example, health professionals employ informal triage systems for a fixed number of HD slots that prioritize clinical acuity, patient age, prior use of CAPD, and place of residence. Such attempts at formalizing medical decision-making in the context of scarcity have been described in other low-resource settings [31]. At UNAERC, shortening and spacing apart dialysis reflects a common strategy in resource-constrained settings when RRT resources are insufficient to meet demand [32–34]. Completing paperwork requirements at UNAERC becomes a critical skill to access care, a notion described in other scholarship on access to biomedical institutions in Guatemala [35].

Another example of the clinical-level impact of system-level constraints is that encouraging CAPD over HD emerges as a central strategy to offload an overburdened system. At UNAERC, health professionals perceive that resource limitations are less significant for CAPD than for HD and that patients are likelier to have a good outcome with CAPD. Thus, although there is no official CAPD-first policy, in practice patients are often preferentially guided to CAPD. We are not aware of data in Guatemala comparing the cost of HD vs. CAPD and, globally, cost-effectiveness may depend on country-level factors such as the availability of locally manufactured dialysis supplies [36–38]. In other low-resource settings, explicit CAPD-first programs have been implemented to take advantage of CAPD’s technical simplicity, minimal infrastructure requirements, and capability to be carried out in isolated geographic areas [38, 39]. Importantly, the prioritization of CAPD at institutions like UNAERC is not an example of scaling a cheaper though clinically inferior treatment [40], as HD and CAPD are both accepted dialysis modalities with generally similar clinical effectiveness [41, 42]. At the same time, the use of an informal rather than formal CAPD-first policy may feel coercive to some patients, particularly since prior CAPD patients are prioritized for limited HD slots.

Second, a significant finding of our study was the degree to which dedicated, highly skilled health professionals at UNAERC suffered work-related emotional challenges. Central to feelings of shame, disempowerment, and sadness were interviewees’ perceptions that they could not offer patients standard-of-care treatments. This finding aligns with global literature in which front-line clinicians experience distress when they allocate dialysis and other costly medical technologies [33]. In one study in Nigerian public hospitals, a majority of specialist physicians were so bothered by bedside rationing that they regretted the decision to enter the field of medicine [43]. In South Africa, an explicit dialysis rationing strategy caused distress among clinicians who viewed dialysis provision as driven by socioeconomic factors like poverty and race rather than objective clinical factors [44, 45].

In our study, respondents described their emotional experience using the term “burnout.” Burnout is a job-related condition of chronic exhaustion first conceptualized in the U.S. clinical workforce in the 1970s and since applied to other occupations and countries [46, 47]. A commonly cited definition refers to burnout as “a syndrome of emotional exhaustion and cynicism that occurs frequently among individuals who do ‘people-work’ of some kind” [48]. Burnout and its adverse consequences previously have been described among health professionals in Guatemala [49–51] and in dialysis units in other settings [52, 53]. Notably, health professionals at UNAERC described the experience of burnout alongside profound sentiments of solidarity, purpose, and satisfaction in serving patients.

Yet, it is not clear from our interviews whether burnout best captures our respondents’ emotional experiences, which may be better understood through the concept of moral distress. Moral distress arises “when one knows the right thing to do, but institutional constraints make it nearly impossible to pursue the right course of action [54].” Originating in the nursing literature, moral distress emphasizes structural factors that predict the health of patients and the wellness of clinicians [55]. As Armin articulates, “the production of moral distress occurs when individuals shoulder the responsibility for rationing health care in the context of public policies that construct deserving and undeserving patients” [56]. An example of moral distress described in the global workforce was the emotional experiences of local African health workers during the Ebola epidemic [57]. At UNAERC, our data call for more resources to support health workers’ wellness and mental health. Additionally, formalizing and transparently communicating triage policies might have the double benefit of attenuating health workers sentiments of moral distress and improving patients’ perceptions of fairness.

Third, respondents at UNAERC suggested various strategies to improve public-sector kidney care in Guatemala, including increasing budgets for equipment and personnel, investing in preventative services, and decentralizing services. Globally, the nephrology community has developed messages to help national actors advocate for investments in ESKD care [58]. One proposed strategy is the implementation of RRT registries to showcase inequitable access and provide data to support proposals for treatment expansion. In Guatemala, an RRT registry was launched in early 2019 [59] that will give even more complete information than previous national studies [12, 16, 60]. These data should be used by UNAERC and patient advocates to inform policymakers and government officials of the burden of CKD and needs of patients with CKD in Guatemala. As a complement to registry data, future research is need on ESKD patients who choose not to access dialysis and the drivers of these decisions [61].

Another path forward in Guatemala, as multiple respondents suggested, would be the expansion of transplantation services. Kidney transplantation is the RRT modality that offers the best survival and quality of life [62]. In Guatemala, renal transplantation has been available for decades, and more than 100 patients are successfully transplanted per year in the country [63, 64]. However, uncertain access to immunosuppressive medications and barriers to care coordination limit access to transplantation in uninsured patients at UNAERC [65, 66].

As our interviewees assert, prevention of CKD in Guatemala also will be critical in future years to attenuate the RRT caseload at UNAERC. While CKDu receives much international attention [15], regional data suggest that traditional risk factors like hypertension and, especially, uncontrolled diabetes are the major drivers of kidney disease [12, 13]. Both of these conditions have evidence-based, low-cost interventions that can prevent CKD onset and progression. However, the political will for strengthening chronic disease care in the primary health system is uncertain.

Limitations of this study include our non-probabilistic sampling technique, inclusion of one nephrology institution in a single country, and a sample of participants that did not include CKD patients. First, we justify our sampling as appropriate within the complex political environment of kidney care in Guatemala where earning the trust of participants was crucial to gain access for interviews. We achieved our sampling goal of generating thematic saturation of perspectives from diverse health professionals rather than physicians or nurses alone. Second, challenges in the delivery of kidney care likely differ at other nephrology centers both within Guatemala—including UNAERC’s satellite site in the southern Pacific zone—and also in other countries. However, our goal was to understand the challenges of providing kidney care to a large number of uninsured adults in Guatemala, and UNAERC is the dominant institution providing such care. Third, while this study did not include patient perspectives, it complements the authors’ prior work studying CKD in Guatemala from the perspective of both children and adults [10, 25, 26, 67, 68].

## Conclusion

Health professionals at a public-sector nephrology center in Guatemala deliver kidney care including dialysis to a large volume of poor, underserved patients. Due to systems-level limitations, health professionals make difficult choices on the stewardship of resources, choices that are linked to emotional challenges including burnout and moral distress. This study offers important lessons in Guatemala and other countries seeking to build capacity to scale-up kidney care.

## Supplementary information

**Additional file 1.** Interview Guide with Administrators and Providers.

## Data Availability

In order to protect participants’ identities, interview transcripts from this study are not publicly available. Datasets from this study are available from the corresponding author on reasonable request. The study’s codebook and coding frequencies are available through Dataverse at https://dataverse.harvard.edu/dataset.xhtml?persistentId=doi%3A10.7910%2FDVN%2FJRFVTJ.

## Abbreviations

CAPD: Continuous ambulatory peritoneal dialysis
CKD: Chronic kidney disease
CKDu: Chronic kidney disease of unknown etiology
ESKD: End-stage kidney disease
FUNDANIER: Fundación para el Niño Enfermo Renal (Foundation for Children with Renal Disease)
HD: Hemodialysis
IGSS: Instituto guatemalteco de seguridad social (Social Security Health System)
LMICs: Low- and middle-income countries
RRT: Renal replacement therapy
UNAERC: Unidad nacional de atención al enfermo renal crónico (National Center for Chronic Renal Disease)
