# Supplementary material for: Challenges in the provision of kidney care at the largest public nephrology center in Guatemala: a qualitative study with health professionals
Source: BMC Nephrol. 2020 Feb 28;21:71. doi: 10.1186/s12882-020-01732-w (PMC7049202; doi:10.1186/s12882-020-01732-w)
Supplement: Supplementary file 1 — Additional file 1. Interview Guide with Administrators and Providers. [file 12882_2020_1732_MOESM1_ESM.docx]

**Additional file 1.** Interview Guide with Administrators and Providers

1. Please tell me about the work you do at UNAERC.
2. Please tell me about the patient population that comes here for services.
3. What are the barriers that patients face to accessing care here?
4. What are some of the challenges you face in your work here?
5. How does the demand for services here at UNAERC compare with the resources available?
6. Do you think there is a need to expand UNAERC services?
7. What sorts of resources would help this institution in general?
8. How do you think access to dialysis services could be improved in

Guatemala?

1. Is there anything else you would like to add?
